# Role of Surface Chemistry in Protein Remodeling at the Cell-Material Interface

**DOI:** 10.1371/journal.pone.0019610

**Published:** 2011-05-09

**Authors:** Virginia Llopis-Hernández, Patricia Rico, José Ballester-Beltrán, David Moratal, Manuel Salmerón-Sánchez

**Affiliations:** 1 Center for Biomaterials and Tissue Engineering, Universidad Politécnica de Valencia, Valencia, Spain; 2 CIBER de Bioingeniería, Biomateriales y Nanomedicina (CIBER-BBN), Valencia, Spain; 3 Regenerative Medicine Unit, Centro de Investigación Príncipe Felipe, Valencia, Spain; University of California Merced, United States of America

## Abstract

**Background:**

The cell-material interaction is a complex bi-directional and dynamic process that mimics to a certain extent the natural interactions of cells with the extracellular matrix. Cells tend to adhere and rearrange adsorbed extracellular matrix (ECM) proteins on the material surface in a fibril-like pattern. Afterwards, the ECM undergoes proteolytic degradation, which is a mechanism for the removal of the excess ECM usually approximated with remodeling. ECM remodeling is a dynamic process that consists of two opposite events: assembly and degradation.

**Methodology/Principal Findings:**

This work investigates matrix protein dynamics on mixed self-assembled monolayers (SAMs) of –OH and –CH_3_ terminated alkanethiols. SAMs assembled on gold are highly ordered organic surfaces able to provide different chemical functionalities and well-controlled surface properties. Fibronectin (FN) was adsorbed on the different surfaces and quantified in terms of the adsorbed surface density, distribution and conformation. Initial cell adhesion and signaling on FN-coated SAMs were characterized via the formation of focal adhesions, integrin expression and phosphorylation of FAKs. Afterwards, the reorganization and secretion of FN was assessed. Finally, matrix degradation was followed via the expression of matrix metalloproteinases MMP2 and MMP9 and correlated with Runx2 levels. We show that matrix degradation at the cell material interface depends on surface chemistry in MMP-dependent way.

**Conclusions/Significance:**

This work provides a broad overview of matrix remodeling at the cell-material interface, establishing correlations between surface chemistry, FN adsorption, cell adhesion and signaling, matrix reorganization and degradation. The reported findings improve our understanding of the role of surface chemistry as a key parameter in the design of new biomaterials. It demonstrates the ability of surface chemistry to direct proteolytic routes at the cell-material interface, which gains a distinct bioengineering interest as a new tool to trigger matrix degradation in different biomedical applications.

## Introduction

The interaction of cells with foreign materials takes place via the adsorbed layer of proteins such as fibronectin (FN), vitronectin, and fibrinogen, representing the soluble matrix proteins in the biological fluids [1]. Cells primarily interact with these proteins via integrins, a family of transmembrane cell adhesion receptors [2]. Integrin-mediated adhesion is a complex process that involves integrin association with the actin cytoskeleton and clustering into focal adhesions: supramolecular complexes that contain structural proteins (vinculin, talin, tensin, etc.) and signaling molecules (focal adhesion kinase – FAK, etc.) [2], [3]. FAK is a nonreceptor protein-tyrosine kinase that becomes activated in response to cell-matrix adhesion. FAK is a key signaling protein contributing to integrin control of cell motility, invasion, survival, and proliferation [4].

The cell-material interaction is a complex bi-directional and dynamic process that mimics to a certain extent the natural interactions of cells with the extracellular matrix [5], [6]. Cells in the tissues are constantly accepting information from their environment from cues in the extracellular matrix ECM [7] and, at the same time, cells are producing and frequently remodeling their matrix [1], [2], [8]. Therefore, it is not surprising that many cells cannot adapt and poorly survive *in vitro* and, conversely, when a foreign material is implanted in the body, the adjacent tissue cells do not interact properly because of lack of their ECM.

A line of previous investigations has shown that cells tend to rearrange adsorbed matrix proteins at the material interface, such as FN, fibrinogen and collagen [9]–[11], in a fibril-like pattern. Using model surfaces – mostly self-assembled monolayers (SAMs) – it has been shown that this cellular activity is abundantly dependent on the surface properties of materials, such as wettability [9], surface chemistry and charge [12]. This evidence raises the possibility that tissue compatibility of such materials may be connected with the allowance of cells to remodel surface associated proteins presumably as an attempt to form their own matrix. Much is known about the interactions between different ECM proteins, but surprisingly less is our knowledge about the ECM composition, organization, and stability at the materials interface.

ECM remodeling is a dynamic process which consists of two opposite events: assembly and degradation. These processes are mostly active during development and regeneration of tissues but, when miss-regulated, can contribute to diseases such as atherosclerosis, fibrosis, ischemic injury and cancer [13]–[16]. The proteolytic cleavage of ECM components represents a main mechanism for ECM degradation and removal [17], [18]. The major enzymes that degrade ECM and cell surface associated proteins are matrix metalloproteinases (MMPs). MMPs are a family (24 members) of zinc dependent endopeptidases, which together with adamalysin-related membrane proteinases that contain disintegrin and metalloproteinase domains (ADAMs or MDCs), such as thrombin, tissue plasminogen activator (tPA), urokinase (uPA) and plasmin are involved in the degradation of ECM proteins. MMPs are either secreted or anchored to the cell membrane by a transmembrane domain or by their ability to bind directly uPA receptor (uPAR) and integrin α_v_β_3_ [19].

The role of MMPs in both development and diseases has been recently extensively studied and reviewed [20] because it is tightly linked with the mechanisms for tumor invasion and metastasis [18]. Also, MMPs regulate cell behavior through finely tuned and tightly controlled proteolytic processing of a large variety of signaling molecules that can also trigger beneficial effects in disease resolution [21].

This work investigates matrix protein dynamics on FN-coated mixed self-assembled monolayers (SAMs) of –OH and –CH_3_ terminated alkanethiols, which constitute an excellent model to vary surface wettability in a broad range while maintaining controlled and simple surface chemistry. SAMs are model organic surfaces that provide defined chemical functionalities and well-controlled surface properties [22], [23]. FN adsorption was investigated (adsorbed surface density, distribution and conformation) and correlated to cell behavior. Cell adhesion and signaling on FN-coated SAMs were characterized via the formation of focal adhesions, integrin expression and phosphorylation of FAKs. The reorganization and secretion of FN was linked to the activity of FN after adsorption on the different chemistries. Finally, the expression (gene and protein) of MMP2 and MMP9 metalloproteinases was used to follow matrix degradation. This work provides a broad overview of matrix remodeling at the cell-material interface, establishing correlations between surface chemistry, FN adsorption, cell adhesion and signaling, matrix reorganization and degradation.

## Results

### Fibronectin adsorption

**Figure 1:**
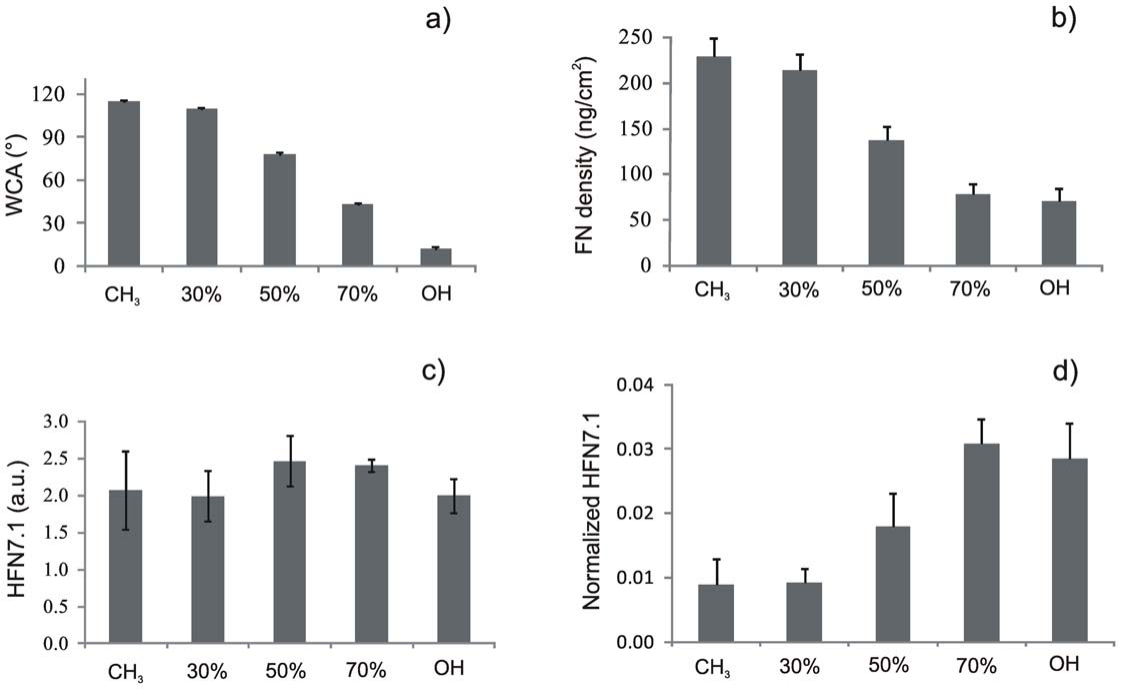
Surface wettability and FN adsorption on the CH_3_/OH mixed SAMs. The horizontal axis displays the percentage of OH groups in SAMs. a) Water contact angle on the different SAMs. b) FN surface density after adsorption from a solution of concentration 20 µg/mL. c) Monoclonal antibody binding for HFN7.1 on the different SAMs after FN adsorption from a solution of concentration 20 µg/mL. d) Activity of the adsorbed FN on the different SAMs obtained by normalizing the monoclonal antibody binding for HFN7.1 relative to the FN surface density calculated in b).

The SAMs prepared in this work have been extensively used and characterized in previous studies making use of XPS, FTIR and ellipsometry [24], [25]. As a routine control, we have measured the water contact angle (WCA) to assess that is in accordance with published results. WCA decreases as the fraction of hydroxy groups increases from 115° on the methyl terminated SAM to 20° on the hydroxyl terminated one (Figure 1a).

The surface density of adsorbed FN was quantified by western blot analyzing the amount of protein remaining in the supernatant after adsorption on the material surface. A calibration curve was built loading gels with known amounts of FN and the resulting bands were quantified by image analysis making use of the Otsu's algorithm to systematically identify the band borders [26]. Each experiment of FN adsorption on SAMs included the loading in the gel of two known amounts of FN (reference points) that correspond to points included in the calibration curve so that the position of the whole calibration curve could be verified for each adsorption experiment [26]. Figure 1b shows the surface density of FN on the different SAMs after adsorption from a solution of concentration 20 µg/mL. The amount of adsorbed protein diminishes monotonically as the –OH density increases from 225 ng/cm^2^ on the methyl terminated SAM to 50 ng/cm^2^ on the hydroxyl terminated one.

The availability of the cell adhesion domains in the adsorbed FN was evaluated by ELISA with monoclonal antibodies, which is a well established method to probe for structural or conformational changes in adsorbed proteins [27], [28]. The antibody used (HFN7.1) was directed against the flexible linker between the 9^th^ and 10^th^ type III repeats of FN [29]. It has been previously demonstrated that HFN7.1 is a receptor-mimetic probe for integrin binding and cell adhesion [29]. HFN7.1 antibody binding is similar on the different SAMs regardless the composition of the surface after FN adsorption from a solution of concentration 20 µg/mL (Figure 1c). However, taking into account that the amount of adsorbed FN differs among SAMs, the availability of the HFN7.1 antibody was obtained by normalizing to the total amount of adsorbed FN on each surface (Figure 1d). This magnitude increases as the fraction of hydroxyl groups on the surface does.

**Figure 2:**
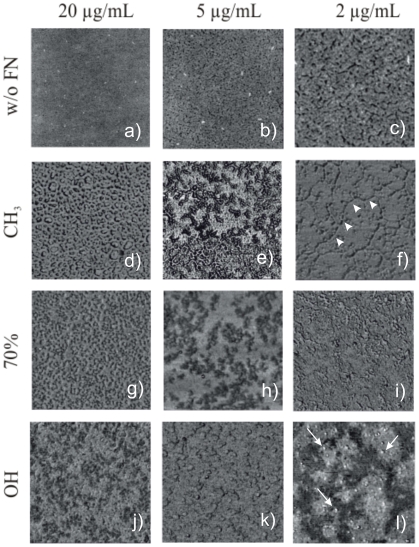
Fibronectin distribution on the different SAMs as observed by the phase magnitude in AFM. The protein was adsorbed for 10 min from different solutions of concentration 20 µg/mL, 5 µg/mL and 2 µg/mL. The first row is the SAM surface without any FN at different magnifications: 5 µm (a), 2 µm (b) and 1 µm (c). Arrowheads in f) identify one of the FN fibers assembled on the material surface upon adsorption (fiber diameter 13±5 nm), arrows in l) identify globular aggregates of molecular size (diameter 20±4 nm). Images including FN are 1 µm side.

The molecular distribution of FN upon adsorption on the different SAMs can be obtained by AFM. Figure 2 shows the organization of FN on three of the surfaces (CH_3_, OH and the surface with 70% OH, that display qualitatively different WCA) after FN adsorption from solutions of different concentrations. FN fibrils are found on the methyl-terminated SAM after adsorption from a solution of 2 µg/mL (average thickness of the fiber is approximately 13±5 nm), less organized molecules are observed on the 70% OH surface that became isolated globular-like molecules on the hydroxyl terminated SAM (average size of the globular aggregates 20±4 nm). Increasing the concentration of the FN solution results in a dense network-like structure of FN on the methyl terminated surface and large molecular aggregates that cover the whole surface for the more hydrophilic surfaces (Figure 2). Figures S1, S2, S3 show AFM images for FN adsorption on the different substrates at different magnifications for the sake of completeness. The fibrillar nature of the adsorbed FN on the methyl-terminated SAM and the globular distribution on the other two surfaces is clearly grasped from this Figures S1, S2, S3.

### Cell adhesion and signaling

**Figure 3:**
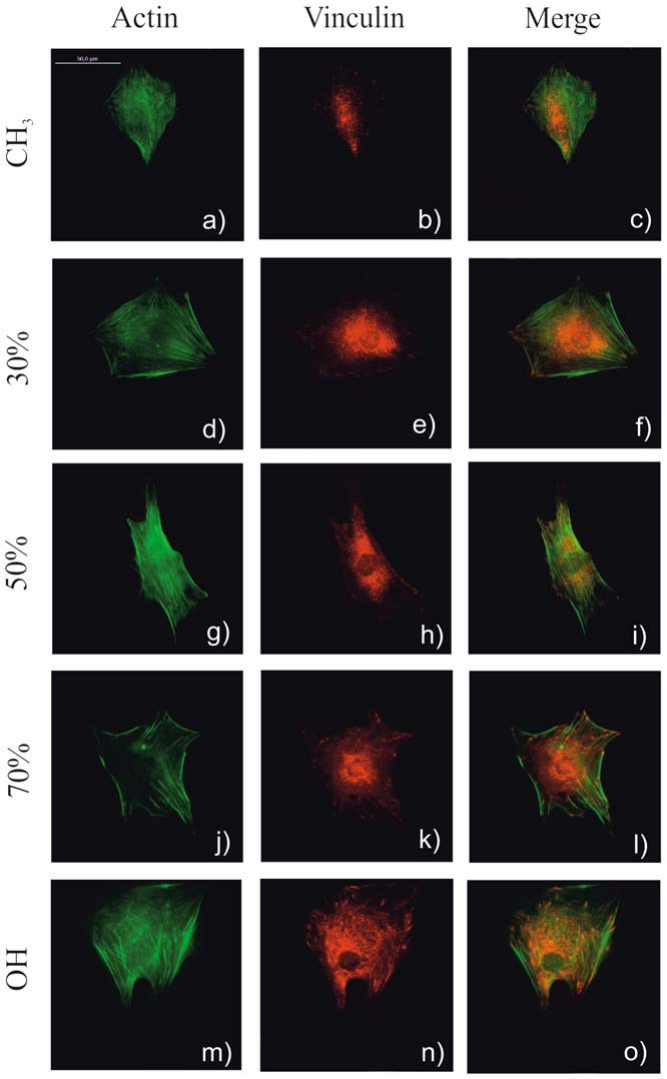
Adhesion of MC3T3-E1 cells after 3 hours on FN coated SAMs. To identify each SAM the percentage of OH groups has been used. First column shows F-actin cytoskeleton, second one the distribution of focal adhesion protein vinculin and its incorporation into focal contact plaques, which is enhanced as the fraction of OH groups increases (see e.g. peripheral organization of well-defined focal contacts in k) and n)). The third column is the superposition of the other two ones. The scale bar in a) is 50 µm.

The organization of proteins involved in the formation of focal adhesion complexes provides an opportunity to learn more about the effectiveness of cell-to-substrate interactions. Figure 3 shows the distribution of vinculin in cells adhering on the different model substrates. Well-defined focal adhesions were found only on the more hydrophilic substrates (OH- terminated and 70% OH). Even if vinculin is expressed also in cells on the more hydrophobic substrates, it is not afterwards organized into focal contacts but randomly distributed throughout the cell. Likewise, the formation of prominent F-actin fibers terminating in well-developed focal adhesion complexes occurs on the hydroxyl-terminated surfaces. More dispersed actin distribution (either lacking stress fiber formation or mostly peripheral staining) is observed as the fraction of OH groups on the surface diminishes (Figure 3).

**Figure 4:**
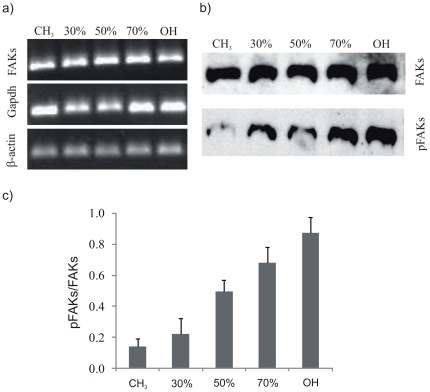
Total FAK expression (protein and gene) and phosphorylation of tyrosine Y-397, the autophosphorylation site in FAK, for MC3T3-E1 cells on FN coated surfaces. SAMs are identified by the percentage of OH groups. a) RT-PCR analysis of FAKs gene expression, β-actin and Gapdh are included as constitutive genes. b) Representative Western blot for total and phophorylated tyrosine residue Y-397 on FAK. c) Quantification of the fraction of phosphorylated FAKs relative to the total FAK expression by image analysis of the western blot bands in b). Error bars represent the standard deviation of three independent experiments; enhanced phosphorylation is obtained as the fraction of OH groups increases.

Focal adhesion kinase (FAK) localizes to focal adhesions to activate multiple signaling pathways that regulate cell migration, survival, proliferation, and differentiation [30]–[34]. We examined the phosphorylation of Y-397, the autophosphorylation site in FAK and a binding site for Src and PI-3 kinases [35], [36]. According to Figure 4 the level of FAK remains constant (both as obtained by analysis of western-blot and PCR bands). By contrast, the ratio between phosphorylated and total FAKs on the different mixed SAMs decreases as the fraction of hydroxyl - terminated groups diminishes (Figure 4c). That is to say, the phosphorylation of specific sites in FAKs depends monotonically on the hydroxyl content of the surface. Likewise, gene expression for FAK as obtained by RT-PCR shows no difference among the different surfaces, while integrin (β_1_) gene expression increases as the fraction of OH on the SAMs does (Figure S4).

### Fibronectin reorganization and secretion

**Figure 5:**
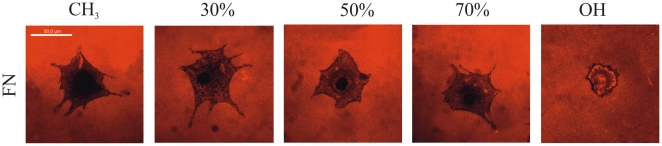
Cellular reorganization of adsorbed FN on the different SAMs after 2.5 h of culture as obtained by immunofluorecence of FN. The red bottom shows FN homogeneously distributed on the material surface. When reorganization of adsorbed FN occurs, black areas (related to the removal of substrate-bound FN) and fibrillar bright areas (as a result of enhanced fluorescence for the incorporation of removed FN into FN-fibrils) are observed. Only the cell shadow in observed for low OH contents (CH3 and 30%). The scale bar represents 50 µm.

Figure 5 shows the cellular reorganization of adsorbed FN after 2.5 h of culture on the different SAMs. It is observed that cells are able to reorganize FN on the hydroxyl-terminated and the 70%-OH SAMs, as it is shown by movements of the adsorbed FN layer with dark zones in the pericellular area, mostly coincident with focal adhesion plaques. Late FN matrix formation was studied for longer times on the different SAMs (Figure S5). It is observed that matrix production increases as time goes by on every substrate. However, cells are able to synthesize and deposit FN matrix more abundantly and better organized into fibrillar networks on the hydroxyl terminated and the 70%-OH SAMs surfaces.

### Matrix degradation

**Figure 6:**
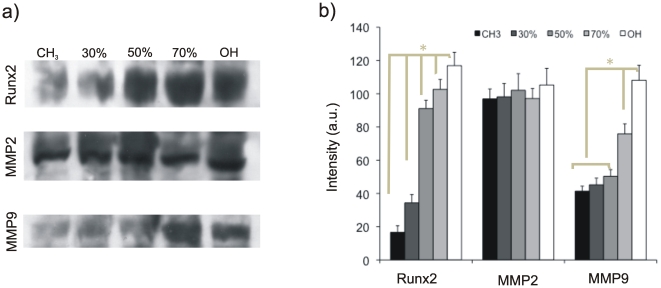
Matrix degradation on the different SAMs quantified by protein expression of matrix metalloproteinases (MMP2, MMP9) and the transcription factor Runx2, which is a target for MMP9. SAMs are identified by the percentage of OH groups. a) Representative Western blot for Runx2, MMP2 and MMP9. b) Quantification of the protein expression by image analysis of the western blot bands. Error bars represent the standard deviation of three independent experiments.

The ability of cells to degrade ECM was investigated by characterizing the expression of two different matrix metalloproteinases (MMPs) and correlated with Runx2 expression. Figure 6 shows characteristic western blot bands for Runx2, MMP2 and MMP9 as well as their relative quantification after 1 day of culture. MMP9 and Runx2 expression increases as the fraction of hydroxyl terminated groups in the surface does. However, MMP2 remain constant regardless the hydroxyl/methyl composition of the material surface.

**Figure 7:**
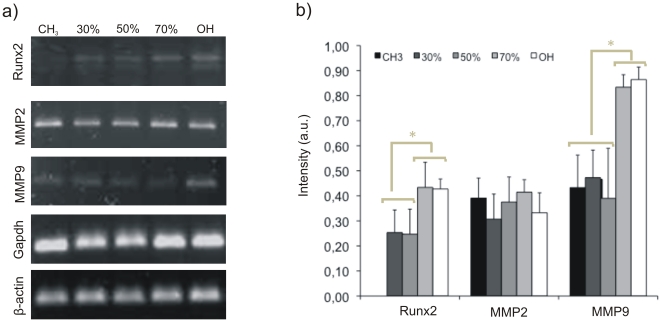
Matrix degradation on the different SAMs quantified by gene expression of matrix metalloproteinases (MMP2, MMP9) and the transcription factor Runx2, which is a target for MMP9. SAMs are identified by the percentage of OH groups. a) Representative RT-PCR bands for Runx2, MMP2 and MMP9; Gapdh and β-actin have been included as constitutive genes. b) Quantification of gene expression by image analysis of RT-PCR bands. The intensity of each band was referred to the level of Gapdh on the same sample. Error bars represent the standard deviation of three independent experiments.

To gain further insights, we investigated gene expression by RT-PCR (Figure 7). Similar levels of MMP2 are found on the different surfaces. By contrast, MMP9 and Runx2 expressions are highly dependent on surface chemistry and with enhanced level on the hydrophilic surfaces. Further, immunofluorescence was used to spatially locate MMP2 and MMP9 during cell culture (Figure S6).

## Discussion

There is a lack of understanding of the cell-material interaction from an integrated point of view that includes the amount and state of the adsorbed layer of proteins on the material surface, cell adhesion - including integrin expression and focal adhesion formation - cell signaling, matrix reorganization, secretion and degradation, i.e. matrix protein dynamics at the cell-material interface. Some efforts have been devoted in the literature to correlate the material surface properties, especially surface chemistry, to protein adsorption and cell adhesion [37]–[40]. Here we present results that provide a link between surface chemistry and cell-mediated matrix protein remodeling (including reorganization, secretion and degradation) on a family of model surfaces (SAMs) with controlled ratio of methyl/hydroxyl groups. From a mechanistic point of view, it is known that the influence of surface chemistry on cell behavior is a consequence of the intermediate layer of proteins adsorbed on the material surface. That is to say, cells interact with synthetic material surfaces via the previously deposited layer of FN. The sequence of events would be the following: FN is a macromolecule that display a globular conformation in solution; upon adsorption on a particular surface chemistry, interactions between the chemical groups of the surface and the FN domains triggers changes in the conformation of the protein that might lead to complete unfolding and exposure of groups that were hidden in solution. Consequently, the effect of the material surface chemistry is indirectly received by cells via the adsorbed layer of FN.

The amount of adsorbed FN on the mixed CH_3_/OH surfaces is lower as the fraction of hydroxyl terminated chains increases (Figure 1b). This is in agreement with results obtained on this family of SAMs by radiolabeling the protein [41]. That is to say, it is known that FN is adsorbed in higher amount on hydrophobic (CH_3_) surfaces than hydrophilic ones (OH) [23]. Our results established the existence of a linear correlation between surface wettability (Figure 1a) and the density of adsorbed FN (Figure 1b) for this family of mixed SAMs. By contrast, the activity of FN after adsorption is higher as the fraction of OH groups on SAMs increased due to the better availability of cell adhesion domains of FN, as it is proved by the HFN7.1 antibody directed to the flexible linker between the 9^th^ and 10^th^ type III repeats of FN [29]. That the activity of FN upon adsorption on SAMs was greater on OH terminated SAMs than CH_3_ terminated ones was previously assessed [23], [42], and our results confirm the finely tuned chemistry-mediated conformation of FN that leads to a monotonically dependence of FN activity on surface composition, as the CH_3_/OH balance on the surface is altered (Figure 1d). It is known that FN has a compact folded structure in physiological buffer that is stabilized through ionic interactions between arms [43]. FN interactions with chemical groups of the substrate (CH_3_) give rise to conformational changes in the molecule that must lead to the occlusion of the cell binding domains (III_9–10_). It is likely that FN orients at the CH_3_ surface, so that its hydrophobic segments interact with the methyl groups in PEA, maybe throughout the heparin-binding fragment [44]. Different supramolecular organization of the protein at the material interface is also reflected in protein distribution on the material surface, as directly observed with AFM images in Figure 2 and Figures S1, S2, S3: globular aggregates on the hydrophilic surfaces and fibrillar-like structures on the methyl terminated SAMs.

Differences in the availability of FN adhesion domains on the different SAMs influence the initial cell-material interaction, as determined by focal adhesion formation and F-actin cytoskeleton development (Figure 3). Gene expression of β_1_ integrin subunit increases with the fraction of OH groups in the sample (Figure S4), which leads to the development of vinculin plaques and actin fibers only on those SAMs on which FN adsorption occurs with the most favorable conformation, i.e. on those chemistries with the highest fraction of OH groups (Figure 3). The influence of surface chemistry on FN conformation and cell adhesion has been established for SAMs based on different chemical groups. In particular, differences in integrin binding and focal adhesion assembly between OH and CH_3_ SAMs most likely resulted from surface chemistry dependent differences in the functional presentation of adsorbed FN, whose major integrin-binding RGD domain is particularly sensitive to the underlying chemistry [41], [45]. Likewise, it was previously found that the number of cells on FBS-coated CH_3_/OH mixed SAMs increases as the fraction of OH groups does; up to 80% OH and then it remains constant [46].

Phosphorylation of FAK has been shown to be sensitive to surface chemistry [45]. In our case, increasing the fraction of hydroxyl groups on the sample leads to similar FAK levels (both for gene and protein expression, Figure 4) but with higher and higher levels of phosphorylation of Y-397, the autophosphorylation site in FAK and a binding site for Src and PI-3 kinases [47], which suggests a stepwise activation of signaling cascades as a function of hydroxyl groups on the surface increases. That is to say, activation of signaling pathways is directly related to integrin binding and focal adhesion formation, which are regulated by the availability of binding domains in FN upon adsorption on different chemistries (Figures 1, 2, 3). It has been demonstrated that FAK regulates cell adhesion strengthening via integrin activation and binding [48]. Moreover, our results are consistent with the role Y-397 autophosphorylation site plays in adhesion strengthening and integrin binding rate. Mutation or blocking of the Y-397 autophosphorylation site blocked FAK-mediated adhesive responses, cell migration and spreading [48]–[51].

After initial cell adhesion, cells tend to reorganize the adsorbed layer of proteins at the material interface before secreting their own matrix. In this way, FN synthesized by cells assembles into a network of fibrils. During this assembly, however, FN needs to undergo distinct conformational changes, which on adsorption to the substrate can be limited. This may explain why materials surfaces affect FN matrix formation [52], [53]. After 2.5 h, cells are able to reorganize the adsorbed layer of FN on the most hydrophilic surfaces (Figure 5) and this ability decreases as the fraction of CH_3_ groups on the surface increases. It has been suggested that the ability of cells to reorganize the adsorbed layer of proteins at the material interface must be a consequence of the strength of interaction between the ECM proteins and the material surface, e.g. materials that bind proteins loosely will support the organization of a provisional ECM [11], [52]–[55]. However, additional reasons must be considered when seeking the molecular origin of this fact, which must also be a consequence of the following sequence of events: i) the availability of cell adhesion domains after FN adsorption on the SAM surface is higher in the samples with higher OH content (Figure 1); ii) integrin expression and focal adhesion formation is enhanced on the more hydrophilic surfaces (Figure 3, Figure S4); iii) phosphorylation of FAK is enhanced on the SAMs with higher OH contents (Figure 4). To reorganize the adsorbed layers of proteins, cells must develop mechanical forces on the substrate through a contractile mechanism. Contractility results from dynamic interactions between actin filaments and myosin, which are regulated via phosphorylation of myosin light chain (MLC). Rho GTPases control the formation of stress fibers and focal adhesion assembly by modulating MLC phosphorylation and generating actin-myosin contractility [56]. It is well known that inhibitors of contractility also down-regulated tyrosine phosphorylation of FAK [57]–[59]; more recently it has been shown that contractility-mediated cell forces also require FAK phosphorylation [60], a fact that supports our reorganization patterns in dependence of the fraction of OH groups: FN is better reorganized on those substrates on which FAK phosphorylation occurs more efficiently (Figures 4, 5).

The dynamics of FN secretion and formation of a fibrillar matrix (late matrix) occurs preferentially on the samples with the higher contents of OH groups (Figure S5); see e.g. the 70%-OH SAM in Figure S5, where the presence of defined FN fibrils of higher fluorescence intensity can be observed. SAMs that promote FN secretion are precisely the substrates on which FN reorganization takes place more intensively (Figure 5). These results support the hypothesis that late matrix formation is in need not only of cell adhesion on the substrate, but some cell movements, in the range of the size of the focal adhesion plaques, must take place so matrix deposition occurs normally [61]. Late matrix formation has been related to the ability of cells to rearrange the initially adsorbed protein layer, especially when comparing cell adhesion on hydrophilic and hydrophobic substrates [52]–[54].

Except organization, the ECM undergoes proteolytic degradation, which is a mechanism for the removal of the excess ECM usually approximated with remodeling. Matrix remodeling is a subject of an extensive biomedical research, but how it relates to the biocompatibility of materials remains unclear. The importance of the proteolytic activity of cells has been already considered in the design of biomaterials by incorporating MMP sensitive sequences, which have shown to be mandatory in tissue regeneration in 3D, including cell proliferation, migration and angiogenesis [62]–[65]. Nevertheless, the effect of material chemistry on the proteolytic activity of cells has not been addressed so far.

Expressions of MMP2 and MMP9 have been observed in MC3T3-E1 cells cultured on tissue culture polystyrene dishes [66]. Our results show that the activation of proteolytic routes in these cells is an MMP-dependent phenomenon sensitive to surface chemistry. MMP2 has FN type II repeats inserted into the catalytic domain [67] and it has been found to cleavage FN and vitronectin into small fragments *in vivo*, which leads to increased cell adhesion and migration [67], [68]. In this sense, MMP2 expression was constant on every FN-coated surface, regardless the underlying chemistry (Figure 6, 7). By contrast, MMP9 expression increases as the fraction of OH groups in the sample does (Figures 6, 7), which suggests a direct relationship between FN activity at the cell-material interface and MMP9 expression, as a consequence of a sequence of events that include integrin expression (Figure S4), focal adhesion formation (Figure 3), matrix reorganization (Figure 5) and FAK phosphorylation (Figure 4). While mechanical strain is known to be able to enhance MMP expression [69], only a few examples in the literature have related the use of synthetic materials on the transcription and activity of MMPs [70]–[73], which we make explicit here by using SAMs with controlled ratio of methyl/hydroxyl groups.

Runx2 is a key transcription factor in regulation of bone development and osteoblast differentiation. The consequence of interfering with endogenous Runx2 is a defect in normal osteoblast development or function [73]. It has been reported a direct relationship between MMP activity and osteblasts markers [74]. In this sense, MMP9 is a direct target of Runx2 in bone tissue, suggesting a regulatory link between Runx2, the expression of MMP9, and cell migration [75], [76]. Figures 6 and 7 also suggest a correlation between Runx2 and MMP9 activation on every surface chemistry. That is to say, Figures 6 and 7 show that both protein and gene expression levels of Runx2 and MMP9 are directly correlated, with low values on the CH_3_-rich SAMs, that increases as the OH content in the surface does. This result supports the idea that surface chemistry-mediated activation of MMP9 occurs in a physiological-like way, as its activation at the cell-material interface involves also the upregulation of its direct target Runx2, as occurs in vivo.

Overall, surface chemistry modulates FN dynamics at the cell-material interface. The ratio CH_3_/OH in mixed SAMs modulates FN adsorption (in terms of the adsorbed density and conformation), cell adhesion (integrin expression and focal adhesion formation), matrix reorganization and secretion. Further, our results demonstrate that surface chemistry is an external parameter able to trigger proteolytic routes in cells in an MMP-dependent manner. Our results demonstrate the ability of synthetic biomaterials as new tools to direct matrix degradation, which must provide the field with new strategies to investigate fundamental aspects of the phenomenon, as well as the inclusion of parameters to take into account during the design of scaffolds for regenerative medicine, aiming at controlling matrix protein dynamics at the cell-material interface.

## Materials and Methods

### Preparation of SAMs

SAM surfaces were prepared and characterized as described elsewhere [23] from alkanethiols 1-dodecanethiol (HS-(CH_2_)_11_-CH_3_), 11-mercapto-1-undecanol (HS-(CH_2_)_11_-OH) (Sigma). Au-coated glass coverslips (Fisher Scientific) were prepared by deposition of thin films of Ti (150 Å) followed by Au (150 Å) using a high vacuum evaporator (Polaron E6100) at a deposition rate of 2 Å/s and a chamber base-pressure of 2·10^−6^ Torr. Glass coverslips were cleaned with 70% H_2_SO_4_ and 30% H_2_O_2_ at room temperature for 1 h, rinsed with deionized H_2_O, rinsed with 95% ethanol, and dried under a stream of N_2_ prior to metal deposition.

Freshly prepared Au-coated surfaces were immersed in alkanethiol solutions (1 mM in absolute ethanol) with different ratios (CH_3_/OH), and SAMs were allowed to assemble overnight. SAMs were rinsed in 95% ethanol, dried under N_2_ and allowed to equilibrate in DPBS prior to incubation in FN solutions. Surfaces were validated by water contact angle measurements (Dataphysics OCA).

### Atomic force microscopy, AFM

AFM experiments were performed using a Multimode AFM equipped with NanoScope IIIa controller from Veeco (Manchester, UK) operating in tapping mode in air; the Nanoscope 5.30r2 software version was used. Si-cantilevers from Veeco (Manchester, UK) were used with force constant of 2.8 N/m and resonance frequency of 75 kHz. The phase signal was set to zero at a frequency 5–10% lower than the resonance one. Drive amplitude was 600 mV and the amplitude setpoint *A_sp_* was 1.8 V. The ratio between the amplitude setpoint and the free amplitude *A_sp_/A_0_* was kept equal to 0.8.

### Protein adsorption

FN from human plasma (Sigma) was adsorbed from solutions of concentrations of 2, 5 and 20 µg/mL in PBS. After adsorption, samples were rinsed in PBS to eliminate the non-adsorbed protein. AFM was performed in the tapping mode immediately after sample preparation.

Separation of FN adsorbed on different samples was performed using 5%-SDS PAGE and denaturing standard conditions as described elsewhere [26]. Proteins were transferred to a PVDF membrane (GE Healthcare) using a semidry transfer cell system (Biorad), and blocked by immersion in 5% skimmed milk in PBS. The blot was incubated with rabbit anti-human FN polyclonal antibody (Sigma, 1∶500) in PBS/0.1% Tween-20/2% skimmed milk for 1 h at room temperature and washed with PBS/0.1% Tween-20. The blot was subsequently incubated in HRP-conjugated secondary antibody (GE Healthcare) diluted 1∶20000 in PBS/0.1% Tween-20/2% skimmed milk. The enhanced chemiluminescence detection system (GE Healthcare) was used prior to exposing the blot to X-ray. Image analysis of the western bands was done using in house software [26].

### Antibody assay for FN conformation

After FN adsorption, surfaces were rinsed in PBS and blocked in 1% BSA/DPBS. Primary monoclonal antibody HFN7.1 (Developmental Hybridoma, Inc., Iowa City, IA) directed against the flexible linker between the 9^th^ and 10^th^ type III repeat was used. Substrates were incubated in primary antibody (1∶4000) for 1 h at 37°C. After washing (0.5% Tween 20/DPBS), substrates were incubated in alkaline phosphatase conjugated secondary antibody (1∶5000) for 1 h at 37°C and incubated in 4-methylumbelliferyl phosphate (4-MUP) (Sigma) for 45 min at 37°C. Reaction products were quantified using a fluorescence plate reader (Victor III, PerkinElmer) at 365 m /465 nm.

### Cell culture

MC3T3-E1 cells were obtained from the RIKEN Cell Bank (Japan). Prior to seeding on FN-coated substrates, cells were maintained in DMEM medium supplemented with 10% foetal bovine serum and 1% penicillin-streptomycin and passaged twice a week using standard procedures. Sample disks placed in a 24-well tissue culture plate were coated with a solution of FN 20 µg/mL. Then, 3·10^3^ cells per substrate were seeded and maintained at 37°C in a humidified atmosphere under 5% CO_2_ for 3 h. Each experiment was performed in triplicate.

### Immunofluorescence (FAKs, MMP, FN)

After 3 h of culture, MC3T3-E1 cells were washed in DPBS (Gibco) and fixed in 10% formalin solution (Sigma) at 4°C. Cells were incubated with permeabilizing buffer (103 g/L sucrose, 2.92 g/L NaCl, 0.6 g/L MgCl2, 4.76 g/L HEPES buffer, 5 mL/L Triton X-100, pH 7.2) for 5 min, blocked in 1% BSA/DPBS and incubated with primary antibody against vinculin (Sigma, 1∶400), MMP2 (abcam, 2 µg/mL;) or MMP9 (abcam, 1∶100). Samples were then rinsed in 0.5% Tween-20/DPBS. Cy3-conjugated secondary antibody in 1% BSA/DPBS (Invitrogen) and BODIPY FL phallacidin (Invitrogen) were used. Finally, samples were washed and mounted in Vectashield containing DAPI (Vector Laboratories). A Leica DM6000B fluorescent microscope was used for cellular imaging.

The ability of cells to reorganize adsorbed FN (i.e., early matrix) was monitored by coating all samples with 20 µg/mL solution prior seeding in serum containing medium. The evolution of FN in the ECM was followed by immunofluorescence after different culture times and following the same procedure as described before. Samples were incubated with anti-FN antibody (1∶400, Sigma) and Cy3-conjugated secondary antibody before washed and mounted with Vectashield containing DAPI.

### Protein expression analysis

Total protein extraction was performed lysing the cells with RIPA buffer (50 mM Tris-HCl pH 7.4, 1% nonidet p-40, 0.25% Na-deoxycholate, 150 mM NaCl and 1 mM EDTA) supplemented with protease inhibitor cocktail tablets (Roche). The lysates were concentrated with Microcon YM-30 Centrifugal Filters units (Millipore) and separated in 7%–10%-SDS PAGE under denaturing conditions. To analyze the different expression patterns of FAKs, p-FAKs, MMPs and Runx2 a conventional Western blot procedure was done as previously described. The blots were incubated separately with primary antibody against FAK (abcam, 400 ng/ml), pFAKs (abcam, 1 µg/mL), MMP2, MMP9 and Runx2 (abcam, 1 µg/mL). In all cases the secondary antibody was HRP linked and the dilutions used were: 1∶50000 for FAKs, 1∶10000 for p-FAKs and 1∶20000 for MMP2, MMP9 and Runx2.

The Supersignal West Femto Maximum Sensitivity Substrate (Pierce) was used prior to exposing the blot to X-ray film.

### Gene expression analysis

Gene expression (mRNA) of β_1_ integrin, Runx2, FAKs, MMP2 and MMP9 was analyzed after 24 h of culture. Total RNA was extracted from cells using RNAeasy Mini Kit (Qiagen). The quantity and integrity of the RNA was measured with NanoDrop (ThermoScientific) and used 3 µg RNA as template for SuperScript III RT (Invitrogen) and oligo(dT)_12–18_ (Invitrogen) as specific primer for amplification of mRNA. PCR reactions were performed with Ampli Taq Gold 360 DNA polymerase (Invitrogen). The oligonucleotides sequence used for PCR reactions are listed in Table 1. All reactions were done at least per triplicate and RNA template was obtained from independent experiments.

**Table 1:** Primer sequences used in gene expression analysis.

Gen	Sequence (5′-3′)	References
β-actin F	TTCTACAATGAGCTGCGTGTG	M_007393.3
β-actin R	GGGGTGTTGAAGGTCTAAA	
Gapdh F	GTGTGAACGGATTTGGCCGT	NM_008084.2
Gadph R	TTGATGTTAGTGGGGTCTCG	
β integrin F	GGAGGAATGTAACACGACTG	[77]
β integrin R	TGCCCACTGCTGACTTAGGAATC	
FAK F	GGAGTTTTCAGGGTCCGACTG	[77]
FAK R	CATTTTCATATACCTTGTCATTGG	
Runx2 F	GTGCTCTAACCACAGTCCATGCAG	NM_001146038.1
Runx2 R	GTCGGTGCGGACCAGTTCGG	
MMP2 F	TGGTGTGGCACCACCGAGGA	NM_008610.2
MMP2 R	GCATCGGGGGAGGGCCCATA	
MMP9 F	AGCACGGCAACGGAGAAGGC	NM_013599.2
MMP9 R	AGCCCAGTGCATGGCCGAAC	

### Statistical analysis

All experiments were performed at least three times in triplicate unless otherwise noted. Data are reported as mean ± standard error. Results were analyzed by one-way ANOVA using SYSTAT 8.0 (SPSS). If treatment level differences were determined to be significant, pair-wise comparisons were performed using a Tukey post hoc test. A 95% confidence level was considered significant.

## Supporting Information

Figure S1 Fibronectin distribution on the different substrates as observed by the phase magnitude in AFM at different magnifications. The protein was adsorbed for 10 min from a solution of concentration 20 µg/mL.
(PDF)

Figure S2 Fibronectin distribution on the different substrates as observed by the phase magnitude in AFM at different magnifications. The protein was adsorbed for 10 min from a solution of concentration 5 µg/mL.
(PDF)

Figure S3 Fibronectin distribution on the different substrates as observed by the phase magnitude in AFM at different magnifications. The protein was adsorbed for 10 min from a solution of concentration 2 µg/mL.
(PDF)

Figure S4 β_1_ integrin expression increases with the percentage of OH groups in SAMs. A) Representative bands for gene expression (RT-PCR) of integrin β_1_. B) Image quantification of RT-PCR bands on the different surfaces.
(PDF)

Figure S5 Cellular reorganization of adsorbed FN and synthesized FN fibrils on the different surfaces after 2.5 h, 5 h, 1 d and 3 d of culture. The technique employed in these figures is immunofluorescence with anti-FN antibody. It is shown the adsorbed FN on the material surface (red bottom) and the way cells rearrange this layer of FN resulting in black-dark areas as well as enhanced intensity of the fluorescence as a consequence of the formation of FN fibrils by cells. It is shown a broad cell population (20–30 cells per image) after different culture times, so that not only FN reorganization is observed but also FN secretion can be accounted for. The adsorbed FN (red bottom) superimposed with cell-secreted FN fibrils on some SAMS (e.g. 70%).
(PDF)

Figure S6 Immunofluorescence for matrix metalloproteinases MMP2 and MMP9 after 1 day of culture on the FN-coated SAMs (identified by the percentage of OH groups). Fluorescence distribution and intensity is in agreement with protein expression displayed in Figure 6. The corresponding image for F-actin is also included for the sake of cell identification. The scale bar is 50 µm.
(PDF)
