# Supplementary material for: Role of Surface Chemistry in Protein Remodeling at the Cell-Material Interface
Source: PLoS One. 2011 May 9;6(5):e19610. doi: 10.1371/journal.pone.0019610 (PMC3090403; doi:10.1371/journal.pone.0019610)
Supplement: Figure S4 — β1 integrin expression increases with the percentage of OH groups in SAMs. A) Representative bands for gene expression (RT-PCR) of integrin β1. B) Image quantification of RT-PCR bands on the different surfaces. (PDF) [file pone.0019610.s004.pdf]

Role of Surface Chemistry in Protein Remodeling at the Cell-Material Interface

Virginia Llopis-Hernández[1]⊥, Patricia Rico[1,2]⊥, José Ballester-Beltrán[1], David Moratal[1], Manuel Salmerón-Sánchez[1,2,3*]

1 Center for Biomaterials and Tissue Engineering, Universidad Politécnica de Valencia, Spain, **2** CIBER de Bioingeniería, Biomateriales y Nanomedicina (CIBER-BBN), Valencia, Spain, **3** Regenerative Medicine Unit, Centro de Investigación Príncipe Felipe, Valencia, Spain

⊥ These two authors contributed equally to this work. [*] Email: masalsan@fis.upv.es

Supplementary Figures

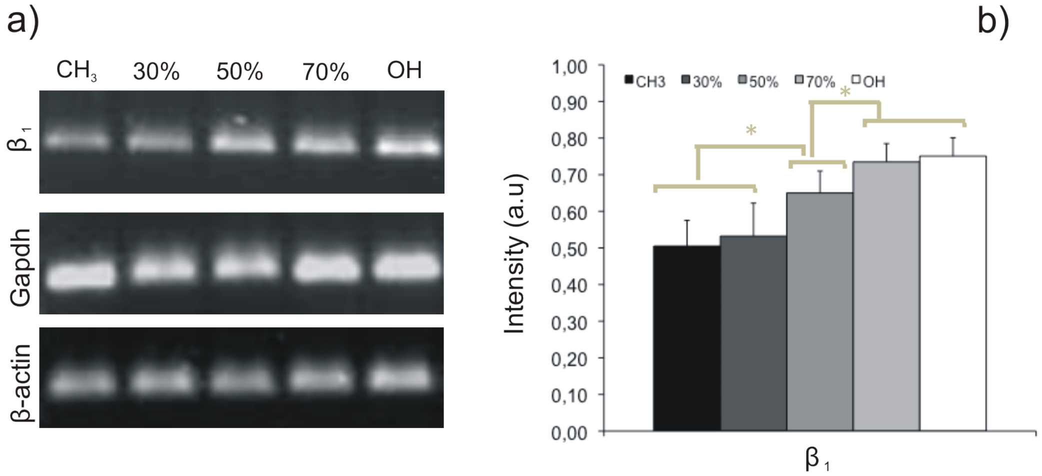

Figure S4. β_1 integrin expression increases with the percentage of OH groups in SAMs. A) Representative bands for gene expression (RT-PCR) of integrin β_1. B) Image quantification of RT-PCR bands on the different surfaces.
