# Supplementary material for: Role of Surface Chemistry in Protein Remodeling at the Cell-Material Interface
Source: PLoS One. 2011 May 9;6(5):e19610. doi: 10.1371/journal.pone.0019610 (PMC3090403; doi:10.1371/journal.pone.0019610)
Supplement: Figure S5 — Cellular reorganization of adsorbed FN and synthesized FN fibrils on the different surfaces after 2.5 h, 5 h, 1 d and 3 d of culture. The technique employed in these figures is immunofluorescence with anti-FN antibody. It is shown the adsorbed FN on the material surface (red bottom) and the way cells rearrange this layer of FN resulting in black-dark areas as well as enhanced intensity of the fluorescence as a consequence of the formation of FN fibrils by cells. It is shown a broad cell population (20–30 cells per image) after different culture times, so that not only FN reorganization is observed but also FN secretion can be accounted for. The adsorbed FN (red bottom) superimposed with cell-secreted FN fibrils on some SAMS (e.g. 70%). (PDF) [file pone.0019610.s005.pdf]

Role of Surface Chemistry in Protein Remodeling at the Cell-Material Interface

Virginia Llopis-Hernández[1]⊥, Patricia Rico[1,2]⊥, José Ballester-Beltrán[1], David Moratal[1], Manuel Salmerón-Sánchez[1,2,3*]

1 Center for Biomaterials and Tissue Engineering, Universidad Politécnica de Valencia, Spain, **2** CIBER de Bioingeniería, Biomateriales y Nanomedicina (CIBER-BBN), Valencia, Spain, **3** Regenerative Medicine Unit, Centro de Investigación Príncipe Felipe, Valencia, Spain

⊥ These two authors contributed equally to this work. [*] Email: masalsan@fis.upv.es

Supplementary Figures

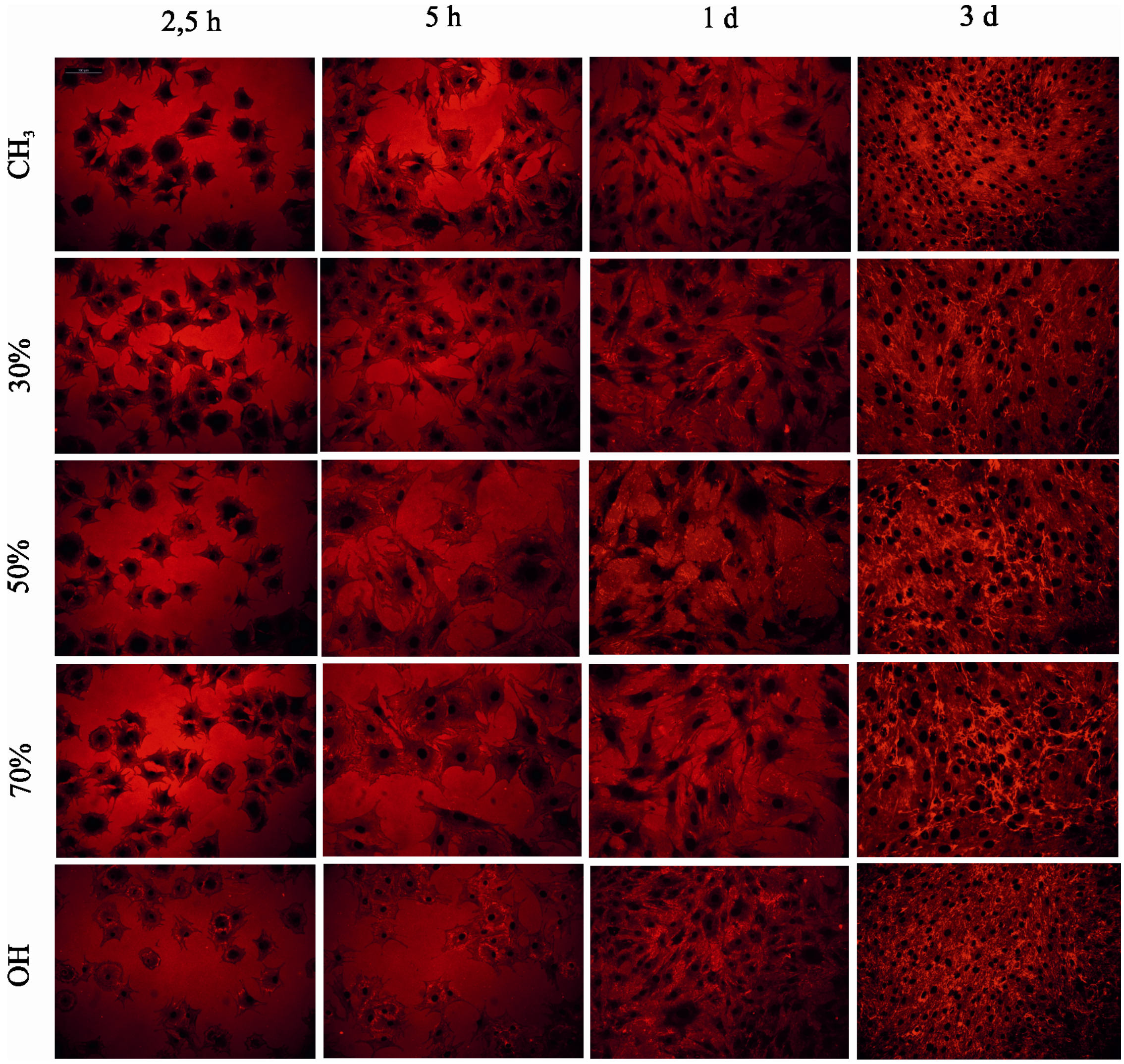

Figure S5. Cellular reorganization of adsorbed FN and synthesized FN fibrils on the different surfaces after 2.5 h, 5 h, 1 d and 3 d of culture. The technique employed in these figures is immunofluorescence with anti-FN antibody. It is shown the adsorbed FN on the material surface (red bottom) and the way cells rearrange this layer of FN resulting in black-dark areas as well as enhanced intensity of the fluorescence as a consequence of the formation of FN fibrils by cells. It is shown a broad cell population (20-30 cells per image) after different culture times, so that not only FN reorganization is observed but also FN secretion can be accounted for. The adsorbed FN (red bottom) superimposed with cell-secreted FN fibrils on some SAMS (e.g. 70%).
